# Colorectal carcinoma presenting as spontaneous colocutaneous fistula - A rare case report and review of literature

**DOI:** 10.1016/j.ijscr.2022.107346

**Published:** 2022-06-24

**Authors:** Sunil Basukala, Yugant Khand, Soumya Pahari, Priya Mainali, Nirvik Gurung, Suman Gurung

**Affiliations:** a Department of Surgery, Shree Birendra Hospital, Chhauni, Kathmandu 44600, Nepal; b Nepalese Army Institute of Health Sciences – College of Medicine, Sanobharyang 44600, Kathmandu, Nepal; c Department of Pathology, Shree Birendra Hospital, Chhauni, Kathmandu 44600, Nepal

**Keywords:** Colorectal carcinoma, Colocutaneous fistula, Right hemicolectomy

## Abstract

**Introduction and importance:**

Colon cancer presenting as spontaneous enterocutaneous fistula are rare with only few cases reported in the literature. Such presentation signifies locally advanced disease with poorer outcomes. Enterocutaneous fistula increases morbidity and mortality in cancer and may potentially delay the definitive care. It poses a difficulty in management in terms of patient optimization, determining the type of resection (palliative or curative) and the operative timeline.

**Case presentation:**

A 47 years old female presented with complaints of foul smelling discharge from a fistulous opening in right iliac fossa with occasional per rectal bleeding for the past six months. Imaging showed ascending colon mass breaching the peritoneum with fistulous tract opening into subcutaneous plane. Exploratory laparotomy with extended right hemicolectomy and en bloc resection was performed.

**Clinical discussion:**

Cutaneous fistula can be caused by traumatic, postoperative etiologies and about 20 % are of spontaneous etiologies. Colon cancer has the ability to mimic any abdominal disease with a wide spectrum of presentations. The locoregional extension from the bowel creates a passage of colonic contents to evacuate from the external opening. The fistulous tract of colon cancer is less likely to close spontaneously and may require surgical intervention following appropriate resuscitation. Due to features suggestive of bowel obstruction an early single stage surgery was performed in our case.

**Conclusion:**

There are no existing guidelines for colon cancer with colocutaneous fistula because they are the same for benign fistulas (resuscitation, control of output, eradication of the infection, nutritional optimization, surgery) along with a multidisciplinary oncology team approach.

## Highlights

• Colon cancers presenting as spontaneous ECF are rare and signifies locally advanced state with poorer outcomes.
• ECF increases morbidity and mortality in cancer and may potentially delay the definitive care.
• Though challenging, surgical resection with curative intent in such case is likely beneficial.

## 1 Introduction

Majority of enterocutaneous fistulas are iatrogenic in origin and occur in the background of surgical complications like enterotomies and intestinal anastomotic dehiscence [1]. Spontaneous ECF comprises about 20 % of ECF [2] and is rare, usually associated with Crohn's disease, diverticulitis, radiation enteritis and malignancy. The mortality of ECF ranges from 15 to 25 % [3] which further increases when associated with an underlying malignancy [4]. In addition to that, presence of ECF may delay the potentially beneficial treatment of the underlying malignancy [5]. The presentation of colonic carcinoma can be diverse, most cases are detected by screening programs while one third can present with emergency complications. ECF is only ever associated with colorectal cancer [6] and when present, it signifies a locally advanced disease. Colon cancers presenting as ECF are rare, only few case reports have been published so far as to our best knowledge [2], [6], [7], [8], [9]. Such presentation of colonic carcinoma poses a difficulty in management in terms of patient optimization, determining the type of resection (palliative vs curative) and the operative timeline. This case is reported in line with the SCARE 2020 guidelines [10].

## 2 Case presentation

A 47 year-old female presented to the emergency department with the complain of foul smelling feculent discharge from right lower abdomen for 2 weeks. It was insidious in onset and was associated with fever, nausea, and reduced appetite. She did not have jaundice nor hematemesis. However, she gave a history of intermittent constipation with occasional per rectal bleeding, abdominal discomfort and sense of incomplete evacuation after defecation for the past six months. She also complained of significant weight loss for the past three months. There was no significant medical and surgical history. There was no family history of colon cancer, colonic polyp or endometrial carcinoma. She does not consume alcohol and is a non-smoker.

**Fig. 1:**
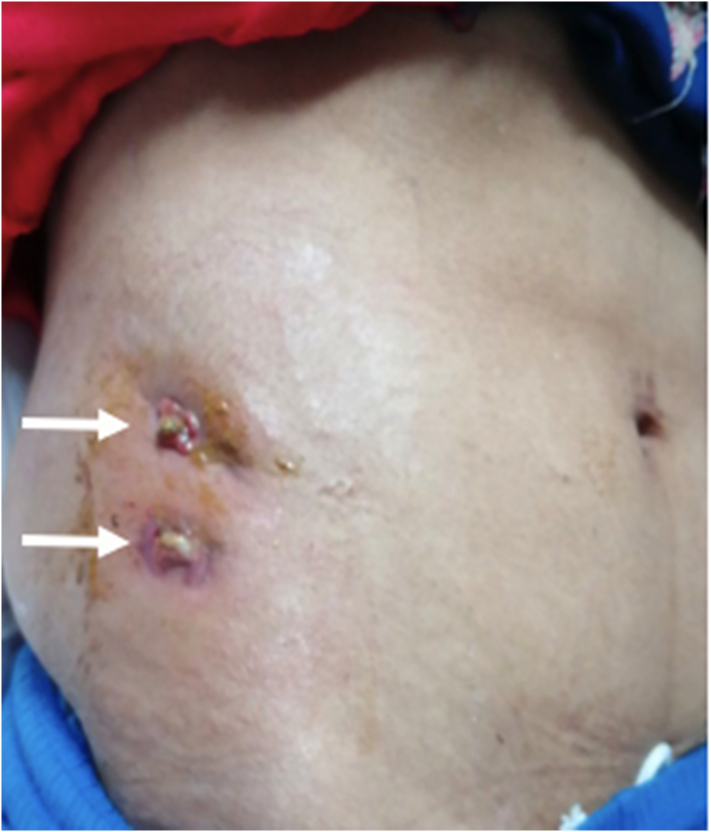
Feculent discharge with two central opening (white arrows) on the anterolateral aspect of the abdominal wall.

On her general examination, she was in acute distress due to pain and was malnourished. Her vital parameters on admission showed she was afebrile, tachycardic and tachypneic. On physical examination, there was oedema of the skin in the right lower abdomen with marked erythema and spontaneous feculent drainage through two anterolateral opening on the anterior abdominal wall Fig. 1. The rest of the abdomen was soft and nontender. On rectal examination, there was a normal tone and the examination finger was stained with stool with no palpable mass. The laboratory investigation revealed an elevated white blood cell count of 14 × 10^3^ per L (normal range 4.0–10.8 × 10^3^ per L) with neutrophilia, hemoglobin was below normal limit at 11 mg/dL (normal range 12–16 g/dL) and albumin 3.1 g/dL (normal range 3.4–4.8 g/dL).

**Fig. 2:**
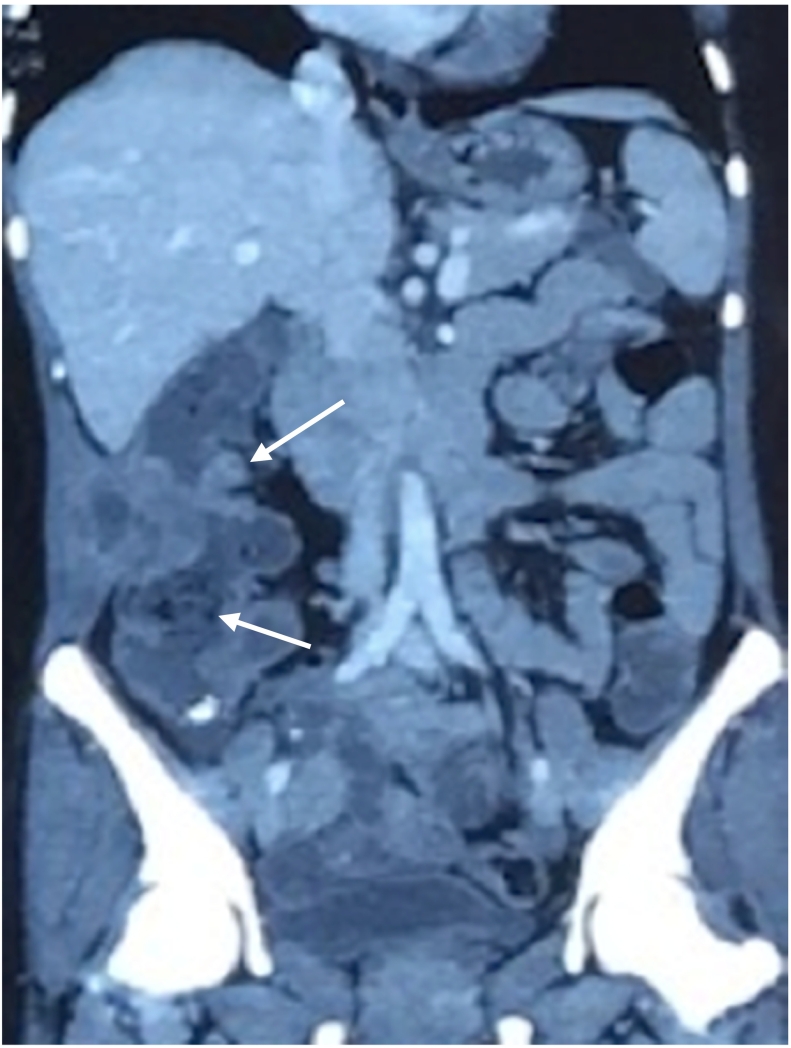
Ascending colon mass with fistulous connection to the anterior abdominal wall shown as arrows.

After resuscitation with intravenous fluids, administration of broad-spectrum intravenous antibiotics and intravenous analgesics, a contrast-enhanced computed tomography (CECT) scan was done to further characterize the lesion. The CECT abdomen showed ascending colon mass extending from the subcutaneous and intermuscular planes over the right iliac fossa and breaching the peritoneum. It showed heterogeneous wall thickening of the proximal part of the ascending colon with loss of mural stratification and causing luminal narrowing, measuring 6.1 × 3.4 × 5.6 cm with surrounding inflammatory changes with presence of a colocutaneous fistula, communicating with the involved section of the ascending colon Fig. 2. The level of carcinoembryonic antigen (CEA) was 7.6 ng/mL.

The procedure was conducted by a team of experienced surgeons. Colonoscopy was planned for the patient but she showed features of bowel obstruction so surgery was performed without bowel preparation. Exploratory laparotomy was performed and a right sided ascending colonic mass approximately 4 × 6 cm in diameter was found adherent to the anterior abdominal wall, with extension through a fistulous tract into the subcutaneous tissue. No distant metastasis was noted intraoperatively.

**Fig. 3:**
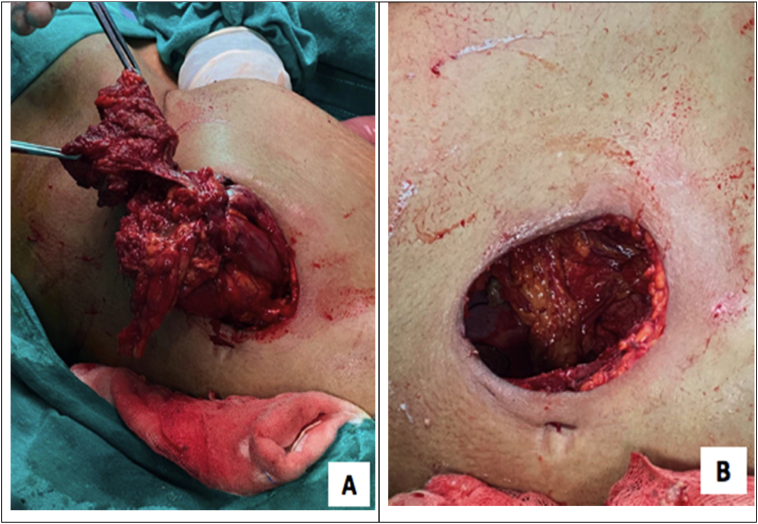
En-bloc resection of ascending colon. A. The tumor with associated mesocolon and colocutaneous fistula and anterior abdominal wall B. Anterior abdominal wall after removal of tumor.

Intraoperative finding showed ascending colonic cancer complicated by colocutaneous fistula. En bloc resection of ascending colon, including the tumor of 4 cm in diameter, was carried out along with the mesocolon and colocutaneous fistula, and the involved anterior abdominal wall around it, according to the standard oncological principles Fig. 3. This was followed by right hemicolectomy and a total of 15 lymph nodes were harvested from the mesocolon. It was followed by en-bloc resection of the involved part in the lateral abdominal wall using a closure technique. The defect was closed primarily and no advancement flap technique was used during the procedure.

**Fig. 4:**
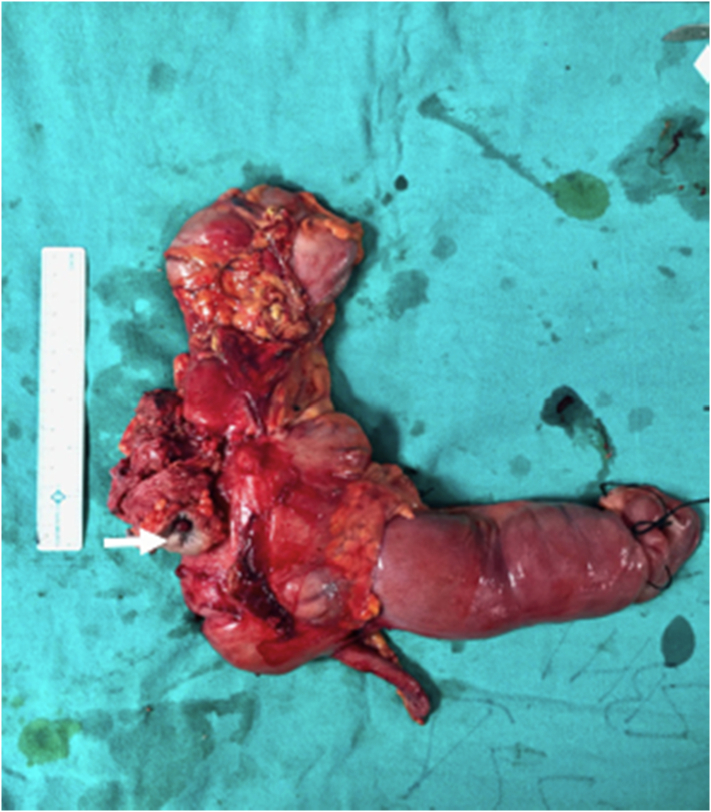
Post-operative specimen showing ascending colon tumor with colocutaneous fistula and anterior abdominal wall (white arrow).

Peritoneal lavage was done and the abdomen was closed with placement of left pelvic drain. Resected specimen showed a whitish circumferential proliferative tumor in the mid-portion of ascending colon Fig. 4.

**Fig. 5:**
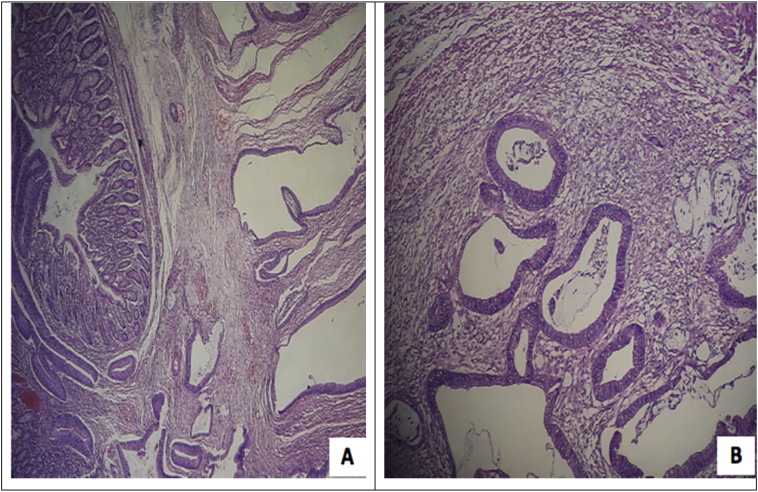
A. Infiltration of tumor cells in a glandular pattern into the submucosa layer. B. Tumor cells forming glands surrounded by desmoplastic reactions.

Histopathology of the resected specimen revealed adenocarcinoma with presence of well-differentiated tumor cells extending into the wall of the fistula and surrounding skin and metastasis in one regional lymph node, classifying it as pT4bN1aM0 Fig. 5.

All respected margins (proximal/distal/radial) of the specimen were free of adenocarcinoma, making it a R0 resection. The patient had an uneventful postoperative recovery. The patient was discharged on the 14th postoperative day with a healthy wound. The patient was referred to the Department of Medical Oncology for further adjuvant therapy. Six cycles of FOLFOX regimen were used during the adjuvant therapy.

## 3 Clinical discussion

Colocutaneous fistula (CCF) is an abnormal connection between the epithelium of the colon and the skin. CCF can be caused by traumatic, postoperative etiologies and about 15–25 % are of spontaneous etiologies [1]. CCF primarily occurs from diverticulitis, inflammatory bowel disease, appendicitis and cancer [11]. The presence of spontaneous cutaneous fistula in colorectal carcinoma (CRC) is a rare entity in itself and denotes a locally advanced stage of cancer.

Colon cancer is the third most common malignancy worldwide and the most common cause of cancer related deaths [12]. Patients with CRC commonly present to the outpatient setting with changes in bowel habit, rectal bleeding or iron deficiency anemia whereas up to one third can present to the emergency department with complications like perforation and obstruction. Colon carcinoma can mimic any abdominal disease with a wide spectrum of presentations [13]. However rare, perforation in CRC may lead to a localized collection intra abdominally and spread superficially into the skin creating a fistulous tract. The usual management of abscesses such as antibiotics or drainage may remain futile owing to the background of malignancy. This locoregional extension from the bowel creates a passage of colonic contents to evacuate from the external skin opening.

CCF is often associated with leakage of bowel contents like frank stool unlike enteric fistula which presents with bilious output. The contents of large bowel and small bowel can be differentiated by identifying bilirubin in the fistulous content. The presence of bilirubin suggests enteric origin. For diagnostic evaluation of stable patients with CCF, abdominal computed tomography (CT) can be performed after 7–10 days following resuscitation and appropriate infection and wound control [14]. Alternatively fistulogram can be performed to determine anatomy of CCF but rarely identifies the specific origin of the tract [15].

The primary approach of CCF is conservative and supportive management. The first line management of CRC includes correcting fluid and electrolyte imbalance, nutritional support, wound care and reducing fistula output. According to a study, CCF spontaneously closes at a range of 10–180 days [16]. However, there may be delay in spontaneous closure in case of distal obstruction and infection or malignancy in the tract [17]. Therefore, fistulous tract of colon cancer will not close spontaneously and ultimately require surgical intervention following appropriate resuscitation. A lag period is allowed prior to definite management of CCF in order to resolve the ongoing sepsis and restore the nutritional status. In most cases, the presence of fistula is indicative of an underlying inflammatory response and early surgical intervention may pose harm due to risk of dense adhesion. A study stated that recurrence rates of postoperative fistula was associated with early surgery and the cause of recurrence was more likely after oversewing than bowel resection [18]. There was a significant mortality ranging 25–50 % in patients with enterocutaneous fistula [4]. The predominant contributor in many cases is considered to be malnutrition [11]. Though the patient was malnourished, timely intervention to treat the fistulous tract allowed reduction in hospital stay of the patient. Adequate nutritional care and infection prophylaxis of the CCF allowed early recovery after surgery. Preoperative diagnosis of an underlying colonic carcinoma is challenging whereas treatment may be incomplete if an inaccurate diagnosis without the recognition of the underlying malignancy [13]. Colon cancer generally requires surgical resection of the tumor with appropriate delay to allow healing of fistulous tract. For patients with a localized fluid collection or abscess, percutaneous drainage can be performed. However, if transabdominal drainage is performed, there is a potential for seeding of the drain tract, and therefore, at the time of definitive resection, the drain tract and rim of the abdominal wall will need to be resected. Similarly, perforation may cause the tumor to adhere to other organs, and an en bloc resection may be necessary in such cases.

Surgical resection with curative intent is likely beneficial and should be considered in all patients who can withstand the procedure [19]. However, achieving R0 resection is difficult in cases of locally advanced colon cancers, requiring larger resection of colon and the adhered abdominal wall which adds to patient's morbidity and mortality. In such cases, neoadjuvant therapy may reduce the size, decrease micrometastasis and make R0 curative resection possible [5]. Role of neoadjuvant therapy in such cases needs to be further explored. The risks and benefits of palliative resection vs initial curative resection following neoadjuvant therapy should be assessed carefully. Owing to the absence of extensive abdominal wall invasion of the tumor with features suggestive of bowel obstruction an early single stage surgery was performed in our case.

## 4 Conclusion

Due to the rarity of such presentations, more cases may be required to formulate formal management guidelines to tackle the associated surgical dilemma and challenges. Lack of patient awareness and screening facilities regarding colon cancer may be the presumable cause. Patients presenting with advanced stages of carcinoma typically have a higher morbidity and mortality, which complicates emergency surgical management.

## Declaration of competing interest

No conflict of interest.

## References

[1] Gribovskaja-Rupp I., Melton G.B. (2016 Jun). Enterocutaneous fistula: proven strategies and updates. Clin. Colon Rectal Surg..

[2] Kumar A., Pahwa H., Srivastava R., Kumar R. (2012). Multiple spontaneous enterocutaneous fistulae on back: a rare presentation of colonic malignancy. BMJ Case Rep..

[3] Heimroth J., Chen E., Sutton E. (2018 Mar 1). Management approaches for Enterocutaneous fistulas. Am. Surg..

[4] González-Avila G., Quezada-Ramírez M.E., Jiménez Pardo E., Bello-Villalobos H. (2005 Apr-Jun). Resultados del tratamiento de fistulas enterocutáneas en pacientes con cancer [Treatment results of enterocutaneous fistulae in patients with cancer]. Rev. Gastroenterol. Mex..

[5] Chamberlain R.S., Kaufman H.L., Danforth D.N. (1998 Dec). Enterocutaneous fistula in cancer patients: etiology, management, outcome, and impact on further treatment. Am. Surg..

[6] Fadaee N., De Clercq S., Fadaee S. (2019). A rare presentation of colonic malignancy: Enterocutaneous fistula secondary to locally advanced cancer. J. Case Rep. Images Surg..

[7] Jethwani U., Bansal A.A., Kandwal V.V. (2013 May 1). Spontaneous enterocutaneous fistula due to colonic malignancy: a rare case report. Arch.Int.Surg..

[8] Wadhwani N., Diwakar D.K. (2018). Localised perforation of locally advanced transverse colon cancer with spontaneous colocutaneous fistula formation: a clinical challenge. BMJ Case Rep..

[9] Bogdanić B., Augustin G., Kekez T., Mijatović D., Hlupić L., Vanek M. (2012 Mar). Perforated ascending colon cancer presenting as colocutaneous fistula with abscess to the anterior abdominal wall at the site of a cholecystectomy scar treated with biologic mesh. Coll. Antropol..

[10] Agha R.A., Franchi T., Sohrabi C., Mathew G., Kerwan A., SCARE Group (2020). The SCARE 2020 guideline: updating consensus Surgical CAse REport (SCARE) guidelines. Int. J. Surg..

[11] Berry S.M., Fischer J.E. (1996). Classification and pathophysiology of enterocutaneous fistulas. Surg. Clin. N. Am..

[12] Arnold M., Sierra M.S., Laversanne M., Soerjomataram I., Jemal A., Bray F. (2017). Global patterns and trends in colorectal cancer incidence and mortality. Gut.

[13] Basukala S., Thapa N., Rayamajhi B.B., Basukala B., Mandal P., Karki B. (2021). A rare presentation of perforated carcinoma of colon as an anterior abdominal wall abscess-a case report and review of literature. J. Surg. Case Rep..

[14] Evenson A.R., Fischer J.E. (2006 Mar). Current management of enterocutaneous fistula. J. Gastrointest. Surg..

[15] Schecter W.P., Hirshberg A., Chang D.S., Harris H.W., Napolitano L.M., Wexner S.D., Dudrick S.J. (2009 Oct). Enteric fistulas: principles of management. J. Am. Coll. Surg..

[16] Martinez J.L., Luque-de-Leon E., Mier J., Blanco-Benavides R., Robledo F. (2008 Mar). Systematic management of postoperative enterocutaneous fistulas: factors related to outcomes. World J. Surg..

[17] Reber H.A., Roberts C., Way L.W., Dunphy J.E. (1978 Oct). Management of external gastrointestinal fistulas. Ann. Surg..

[18] Lynch A.C., Delaney C.P., Senagore A.J., Connor J.T., Remzi F.H., Fazio V.W. (2004 Nov). Clinical outcome and factors predictive of recurrence after enterocutaneous fistula surgery. Ann. Surg..

[19] Baer C., Menon R., Bastawrous S., Bastawrous A. (2017 Jun 1). Emergency presentations of colorectal cancer. Adv. Colorectal Neoplasia.

